# Case report: target antigen and subclass switch in a patient with autoimmune nodopathy

**DOI:** 10.3389/fimmu.2024.1475478

**Published:** 2024-10-07

**Authors:** Luise Appeltshauser, Helena Glenewinkel, Sophia Rohrbacher, Lena Wessely, Carmen Villmann, Claudia Sommer, Kathrin Doppler

**Affiliations:** ^1^ Department of Neurology, University Hospital Würzburg, Würzburg, Germany; ^2^ Neurologische Praxis Dres. Wessely, Menden, Germany; ^3^ Institute of Clinical Neurobiology, University Hospital Würzburg, Würzburg, Germany

**Keywords:** autoimmune nodopathy, node of Ranvier, neuropathy, auto-antibody, contactin-1, biomarker, neurofilament light chain, case report

## Abstract

**Introduction:**

Autoimmune nodopathy (AN) is a new entity in the field of peripheral neuropathies and is defined by the presence of auto-antibodies against structures of the node of Ranvier combined with specific clinico-pathophysiological features and therapy response in affected patients. The target-specific antibodies do not only serve as diagnostic biomarkers but also for treatment evaluation during follow-up.

**Case report:**

We report a 66-year-old female patient with various autoimmune diseases, including a history of membranous glomerulonephritis which presented with acute-onset, sensorimotor tetraparesis, cranial nerve involvement, and mild respiratory insufficiency. Under the suspicion of Guillain–Barré syndrome, she received intravenous immunoglobulins (IVIg) and achieved remission. At 8 months later, she relapsed with now a poor response to IVIg and developed additional features such as severe sensory ataxia, tremor, and neuropathic pain. Anti-contactin-1 IgG2 antibodies were detected, and the diagnosis was reverted to AN. Plasma exchange and rituximab treatment led to a serological remission and corresponding significant clinical improvement, and the therapy was paused. At 2 years after symptom onset, her condition worsened again with sensorimotor symptoms and severe neuropathic pain despite seronegativity for contactin-1. However, serum binding assays to teased nerve fiber staining showed recurring antibody reactivity against paranodal structures. Caspr-1 was identified as a new target antigen via cell-based assay, and high-titer antibodies of the IgG4 subclass were confirmed via ELISA. Hence, a new cycle of plasma exchange and regular rituximab treatment was initiated, with subsequent clinical improvement and serological remission. The serum neurofilament light chain (sNFL) levels were assessed retrospectively and rose and fell together with the antibody titer.

**Discussion:**

This case demonstrates that autoimmunity to (para)nodal structures can reoccur especially in patients prone to autoimmune disorders and can switch its target antigen and subclass in the course of disease. The presence of auto-antibodies against different targets at the node of Ranvier has direct implications for therapeutic management. We suggest a close follow-up of patients with AN after successful therapy. In case of deterioration despite seronegativity, non-specific tests such as teased fiber assays and repeated screening for different target antigens should be considered.

## 1 Introduction

Autoimmune nodopathies (AN) are a new subform of antibody-mediated neuropathies with the node of Ranvier as the site of attack (1, 2). Auto-antibodies are directed against the axonal proteins contactin-1 and its cis-interaction partner contactin-1-associated protein (Caspr-1) and axonal or glial neurofascin isoforms, which are crucial for the maintenance of axoglial adhesion and saltatory conduction (2, 3). The antibodies are pathogenic and lead to an axoglial detachment at the node as well as an impaired protein turnover, resulting in conduction deficits (4–7). Seropositive patients show characteristic features such as a (sub)acute onset, cranial nerve involvement, and sensorimotor paresis, often mimicking Guillain–Barré syndrome (GBS). In the course, additional features like sensory ataxia, tremor, and neuropathic pain may occur, depending on the antibody subtype. Contactin-1 and neurofascin seropositive AN have been associated with nephrotic syndrome and membranous glomerulonephritis (MGN) (8–10). Diagnostic assessment includes tests for antibodies via cell-based assay and confirmation by ELISA or teased fiber immunohistochemistry (1). Testing is crucial as therapy response differs from other immunoneuropathies, and antibody depletion is recommended upon diagnosis. Serum neurofilament light chain levels (sNFL) and the antibody titer can serve as follow-up parameters for the severity and course (7, 11). Here we present a case with a relapse–remitting course and switch of the target antigen and subclass during the course, highlighting the importance of clinico-serological follow-up and diagnostic and therapeutic reevaluation in case of a relapse. In addition to clinical implications, this case of switch of autoantigens may contribute to our understanding of the pathogenesis of the disease, individual susceptibility to paranodal autoimmunity, and possible triggers of a secondary autoimmune disease.

## 2 Methods

### 2.1 Antibody screening

For the detection of auto-antibodies against nodo-paranodal antigens, we performed ELISA, cell-based assay, and teased fibers screening as previously described (12–15). Subclass and titer were assessed via ELISA. For the cell-based assay, we used HEK-293 cells transfected with rat contactin-1, Caspr-1 plasmids, or with both plasmids for contactin-1/Caspr-1 complex analysis and performed double- and triple-immunostaining for serum and commercial antibodies with colocalization analysis. Photomicrographs at 40- and 63-fold magnification were taken using ThunderImager and Software (Leica Microsystems, Wetzlar, Germany).

### 2.2 Neurofilament light chain assessment

The serum NFL levels from different time points were assessed via Ella SimplePlex™ assay and compared to age-matched healthy controls as a reference for normal values as previously described (7).

### 2.3 Clinical data and consent

Clinical data was evaluated from patient’s charts, clinical examinations, and diagnostic tests. Written informed consent was obtained from the patient for the publication of this case report.

## 3 Case description

### 3.1 Symptoms and diagnostic assessment

A 66-year-old female patient with a history of autoimmune disease (psoriatic arthritis and Hashimoto thyroiditis) was admitted to a nephrology department due to nephrotic syndrome with heavy proteinuria (>5 g/day) and leg edema. A kidney biopsy revealed a membranous glomerulonephritis and antibody screening including PLA2R, THSD7A, KELL1, and STEK1 was negative, so the disease was classified as idiopathic. She was treated with prednisolone, cyclophosphamide, and cyclosporin. Concurrently, the patient developed mild polyneuropathy, which was thought as due to the immunosuppressant and not further assessed neurologically. At 14 months later, she developed severe neurological symptoms including double vision, muscle weakness, gait instability, paresthesia, and neuropathic pain. The neurological exam revealed mild proximal, and distal symmetric tetraparesis, cranial nerve involvement with oculomotor palsy and ptosis, severe sensory ataxia with inability to walk, and distal-symmetric multimodal sensory impairment. The cerebrospinal fluid (CSF) exam revealed a cytoalbuminologic dissociation (protein 131 mg/dL and cell count 4/µL). Nerve conduction studies showed increased distal motor latencies (8.4 ms at the median nerve) and F-wave latencies (68 ms at the tibial nerve and 48 ms at the median nerve). She was diagnosed with GBS and treated with 30 g of intravenous immunoglobulins (IVIg; see **Table 1**). As her renal condition had worsened simultaneously (proteinuria, renal insufficiency grade III with creatinine increase, and a second renal biopsy showing active necrosis), she additionally received rituximab (2× 1,000 mg within 14 days), with subsequent continuous nephrological and neurological improvement. Oral prednisolone therapy was continued, and she pursued neurological rehabilitation, with a complete remission of the neurological symptoms.

**Table 1:** Treatments and response to therapy during the course of the disease, along with antibody titers and sNFL levels, if assessed.

Timeline (months)	Treatment	Doses/regimen	Response	Antibody titer (before treatment)	sNFL levels (pg/mL)
-14	Cyclophosphamide, oral prednisolone, cyclosporin	N/A	N/A (nephrologic treatment)	N/A	N/A
0	IVIg	30 g	Good	N/A	N/A
0	Rituximab	2× 1,000 mg in 14 days	Good	N/A	N/A
6	IVIg	30 g	moderate	N/A	N/A
9	Plasma exchange	5×	Very good	Anti-contactin-1 1:500	578
25	IVIg	30 g	Poor	Anti-Caspr-1 1:4,000	N/A
27	Plasma exchange	5×	Poor	Anti-Caspr-1 1:5,000	50.4
28	Rituximab	2× 1,000 mg in 14 days	Good	N/A	N/A
29	Methylprednisolone	3 g	N/A	N/A	N/A
32	Rituximab	1,000 mg	Good	Anti-Caspr-1 1:1,000	718
38	Rituximab	1,000 mg	Good	Seronegative	33.9

Time point “0” is defined as the first onset of GBS-like symptoms.

GBS, Guillain–Barré syndrome; IVIg, intravenous immunoglobulins; N/A, not assessed; sNFL, serum neurofilament light chain; PE, plasma exchange.

At 8 months later, she relapsed. The neurologic workup revealed mild distal-symmetric sensorimotor tetraparesis with sensory ataxia and hyporeflexia as well as cytoalbuminic dissociation in the CSF analysis. Furthermore, she developed acute renal failure (GFR, 30 mL/min). Electroneurography showed a conduction block at the tibial nerve, prolonged F waves (62 ms at the tibial nerve and 44 ms at the median nerve), and increased distal motor latencies (6.7 ms at the median nerve), whereas spontaneous activity or signs of chronic neural damage were absent in electromyography. The patient was diagnosed with recurrent GBS and treated with intravenous immunoglobulins (30 g). Despite therapy, her condition deteriorated further and she developed mild respiratory insufficiency, cranial nerve involvement (double vision and bilateral facial palsy), burning and stinging neuropathic pain, and postural tremor of the hands. At discharge to rehabilitation 2 months later, she was still wheelchair-bound due to proximal and distal symmetric paresis [Medical Research Council (MRC) sum score 51/60] and severe sensory ataxia including the core, accompanied by neuropathic pain requiring opioid treatment.

### 3.2 Diagnosis of contactin-1 IgG2 autoimmune nodopathy

**Figure 1:**
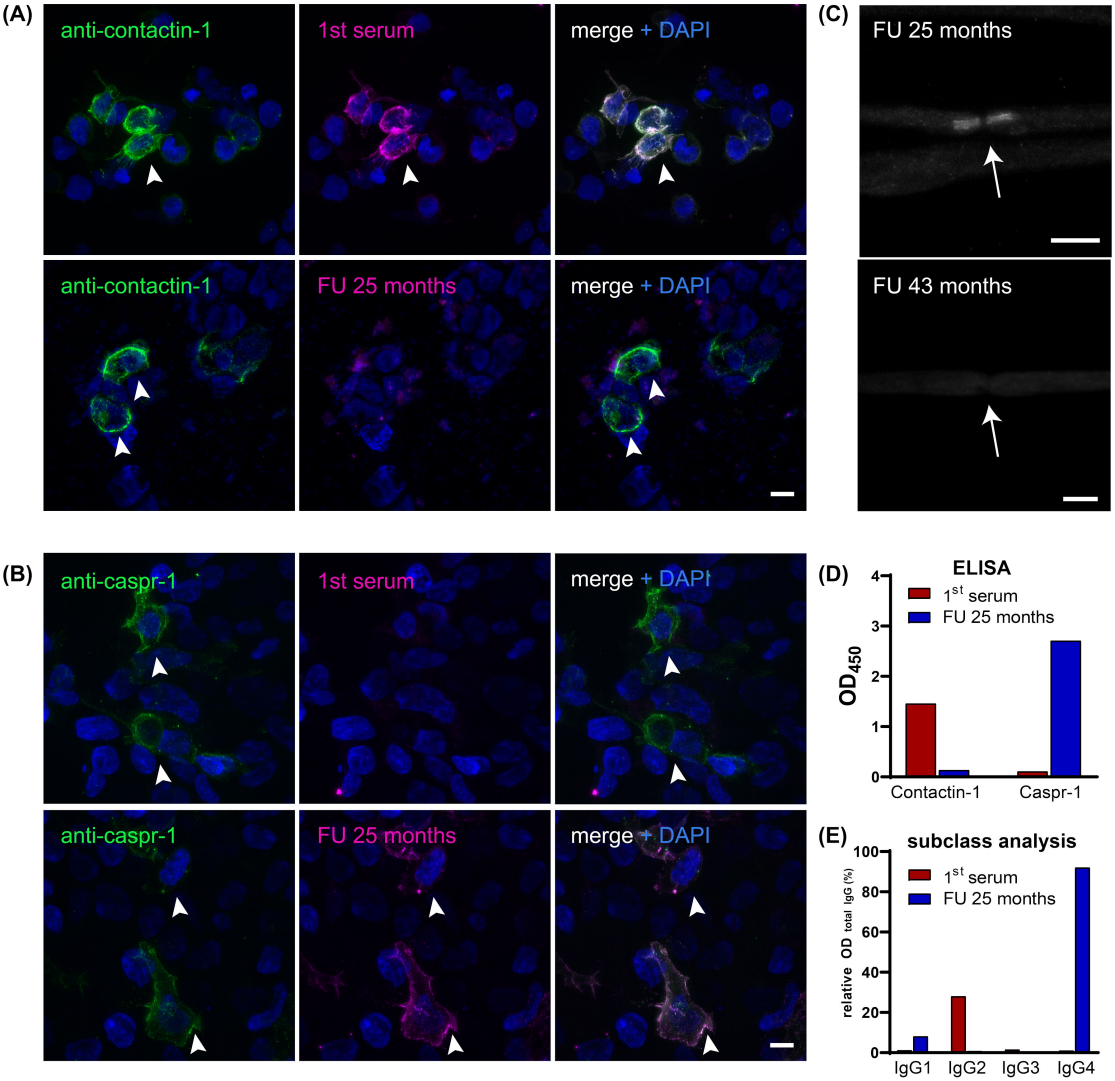
**(A)** Double immunofluorescence staining on contactin-1-transfected HEK-293 cells (arrowheads) with DAPI staining (blue) of both transfected and non-transfected cells, including commercial antibody against contactin-1 (green) and serum (magenta) from different time points (first serum 8 months after symptom onset and follow-up serum at relapse 25 months after the onset). Colocalization appears white and marks a positive result for anti-contactin-1, as present in the first serum, but not at follow-up. Scale bar = 10 µm. **(B)** Double immunofluorescence staining on Caspr-1-transfected HEK-293 cells (arrowheads), including commercial antibody against Caspr-1 (green) and serum (magenta) from different time points (see above). Colocalization of serum and antibody binding only occurs in the follow-up serum, not at onset, showing that anti-Caspr-1 antibodies appeared at relapse during the course of the disease. Scale bar = 10 µm. **(C)** Despite seronegativity for anti-contactin-1, the follow-up serum shows paranodal staining at the node of Ranvier (arrow), indicating reactivity for further paranodal target antigens. After treatment (follow-up serum after 43 months), paranodal staining disappeared, corresponding to seronegativity for both anti-contactin-1 and anti-Caspr-1. Scale bar = 10 µm. **(D)** Bar graphs show optical densities (OD) in the anti-contactin-1 and anti-Caspr-1 ELISA when testing the first serum (red) and follow-up serum (blue). Distinct reactivity gives evidence of a switch of the target antigen from contactin-1 to Caspr-1. **(E)** Subclass analysis is shown relative to the OD of total IgG and reveals predominant IgG2 subclass for anti-contactin-1 and IgG4 subclass for anti-Caspr-1 antibodies. DAPI, 4′,6-diamidino-2-phenylindole; FU, follow-up; OD, optical density.

**Figure 2:**
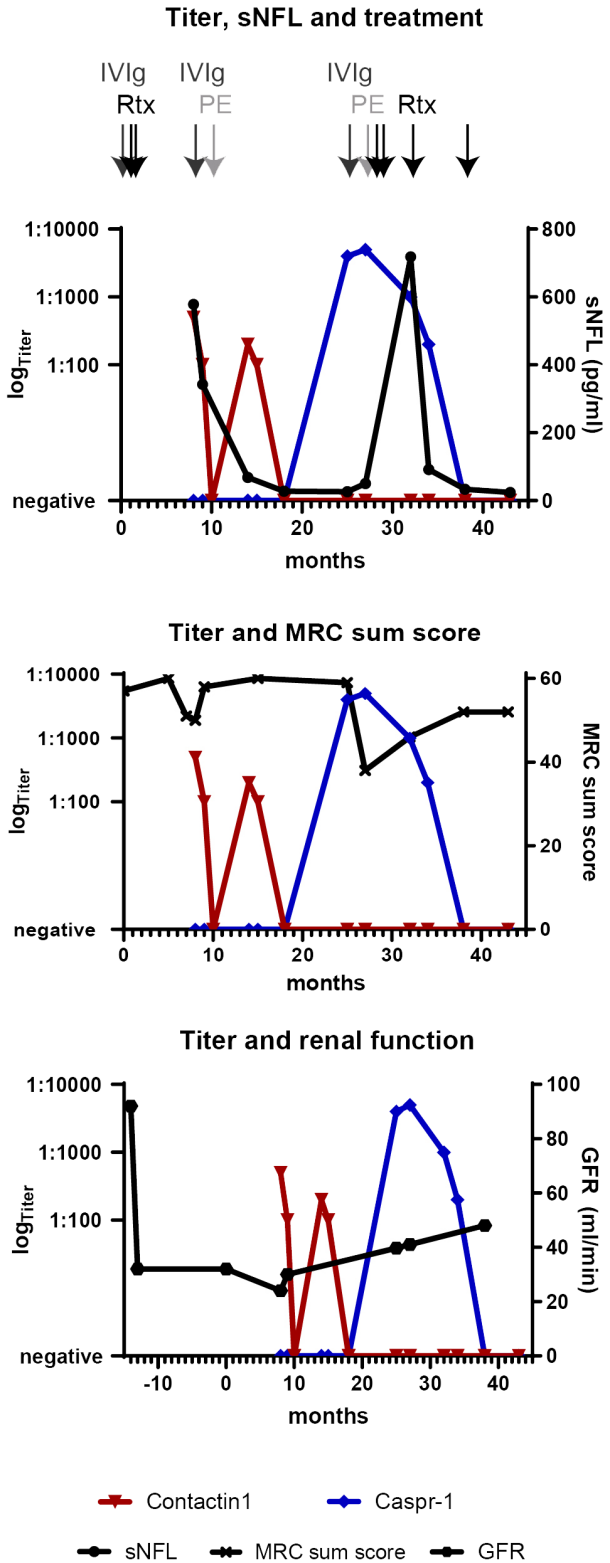
Clinical and serological longitudinal follow-up parameters are shown from 14 months before the onset of neurological symptoms (defined as “0”) to the last follow-up at 43 months (3.5 years). Time points of different immunotherapies are shown by arrows on the top. The titers of anti-contactin-1 (red) and anti-Caspr-1 antibodies (blue) are displayed in a logarithmic scale on the left y-axis. sNFL levels, MRC sum score, and GFR as a marker for renal function (linear, right y-axis) correspond to anti-contactin-1 titer nadirs and improve upon serological remission. After antigen switching, the high anti-Caspr-1 titer is associated with a persistent reduction of the MRC score and highly elevated sNFL in the course, whereas the renal parameters stay stable. Upon serological remission, the sNFL levels have normalized. IVIg, intravenous immunoglobulins; MRC, Medical Research Council; PE, plasma exchange; Rtx, rituximab; sNFL, serum neurofilament light chain.

During rehabilitation, serum was tested positive for anti-contactin-1 antibodies (see **Figure 1A**). A subclass analysis revealed mainly IgG2 (see **Figure 1D**), with a titer of 1:500. The diagnosis was now reverted to autoimmune nodopathy, and she was transferred to a specialized center 10 months after the first occurrence of the neurological symptoms, where she received plasma exchange (PE). Thereby, complete serologic remission could be achieved, accompanied by immediate clinical effects. She reported a complete remission of paresthesia and improvement of muscle strength (MRC sum score 58/60). During subsequent rehabilitation, her symptoms ameliorated further. After several months, she was able to walk 100 m using one walking aid. Anti-contactin-1 antibodies were present at low titer (1:100). At 18 months after onset, she was able to walk without aid for 500 m, with residual distal hypesthesia and minor gait instability. At that time point, anti-contactin-1 antibodies were not detectable anymore (see **Figure 2**) and remained negative in further follow-up testing after 6 months.

### 3.3 Relapse with a switch of the target antigen and subclass

At 25 months after the first episode of the disease, her symptoms relapsed gradually. She now reported proximal muscle weakness, increasing hypesthesia, paresthesia of the face, strong lower back pain, and progressive gait instability. By the decision of the local treating physician, IVIg was administered and had only minor effects. Over several months, her condition deteriorated, and she developed severe proximal and distal tetraparesis (MRC sum score 38/60) and was wheelchair-bound. She was admitted to the hospital for a diagnostic reevaluation. The renal parameters were stable (see **Figure 2**). Electroneurography showed deterioration, with no response when stimulating the tibial nerve. Electromyography revealed signs of chronic neural damage. The serum-anti-contactin-1 levels were re-tested but remained negative. Nevertheless, teased fiber staining showed paranodal staining suggestive of anti-paranodal antibodies (see **Figure 1C**). A further analysis using cell-based assay and ELISA now revealed antibodies against Caspr-1, of subclass IgG4 (see **Figures 1B, D, E**). The titer was highly elevated (1:5,000, see **Figure 2**).

Due to the clinic-serological relapse with target antigen switch to anti-Caspr-1 IgG4, she received plasma exchange (5×), but her state worsened further. Therefore, rituximab (2× 1,000 mg) was applied with good response and slow clinical improvement, followed by two more applications in a 6-month interval. At 1 year after the relapse and switch to anti-Caspr-1, a serological remission was achieved. Upon the last follow-up at 6 months later, seronegativity persisted. As a residue, the patient presented with distal atrophic tetraparesis, drop feet, and gait instability and suffered from persisting neuropathic pain requiring pregabalin treatment. Electroneurography showed signs of distal axonal damage, with no or very low stimulus response on the peroneal and tibial nerves. Electromyography showed distal spontaneous activity and signs of chronic damage. Clinical and serological follow-up examinations will continue every 6 months.

### 3.4 Neurofilament light chain levels corresponded to titer

Serum NFL levels were assessed retrospectively and are shown in **Figure 2**. At diagnosis of AN, sNFL was highly increased (578 pg/ml) but decreased up to normalization during the follow-up, together with the anti-contactin-1 antibody titer. Upon antigen switch to anti-Caspr-1 IgG4, sNFL rose again to high levels, but with a delay and nadir at 6 months after the first symptoms of relapse (718 pg/mL). In further follow-up visits during the 6-month intervals and serological remission, sNFL fell to normal levels (24.6 pg/mL at the last follow-up).

## 4 Discussion

Here we present a long-term follow-up of a case of AN with concomitant MGN and a target antigen and subclass switch during the course of the disease.

This case demonstrates the urge for serological testing for anti-(para)nodal antibodies if “red flags” for AN are present, as recommended by the current guidelines (1, 16). Our patient showed all typical features of contactin-1 and Caspr-1 seropositive AN, such as acute and severe onset of neurological symptoms mimicking GBS, a relapse of symptoms and insufficient response to standard therapy, severe sensory impairment with prominent sensory ataxia, tremor, and neuropathic pain in the course, concomitant MGN, and typical electrophysiological features (1, 12, 17, 18). In the beginning of the disease, the differential diagnosis of GBS and AN can be difficult. In contrast to GBS, AN patients may not respond to standard treatment, may show continuous progressions, or are more likely to relapse (16). Upon suspicion, testing for anti-(para)nodal antibodies should therefore be conducted as early as possible to prevent a delay in therapy.

MGN was initially considered idiopathic but, retrospectively, most likely was associated with anti-contactin-1 antibodies, as in 1% to 2% of cases with MGN (10, 19). Contactin-1 has been defined as a new target antigen in primary MGN (19), and heavy proteinuria has been proposed as a biomarker to distinguish between other immune-mediated neuropathy and AN (8). So far, there have been no reports of Caspr-1 seropositive AN with MGN, and the renal function of our patient remained stable after switching the target antigen. Contactin-1 is expressed in the kidney, but Caspr-1 hardly is (20, 21), possibly explaining the difference in renal pathology. Correspondingly, MGN was present in cases of contactin-1, but not Caspr-1, seropositive patients who underwent subclass switch (22). Thus, patients with MGN and signs of neuropathy should rather be screened for contactin-1 than Caspr-1 antibodies.

Here we report a switch of the target antigen and subclass in the course of AN. In a previous study, we identified another patient with IgG subclass switch from contactin-1/Caspr-1 IgG3 to Caspr-1 IgG4, possibly due to natural, sequential switching and affinity maturation (14). In the case described above, the antibodies did not bind to the contactin-1/Caspr-1 complex, as previously reported (23), but recognized either one or the other protein. Contactin-1 and Caspr-1 belong to different protein families and therefore do not share many common epitopes (3). Hence, the switch from contactin-1 to Caspr-1 is not explained by autoimmunity against complex epitopes or affinity maturation. More likely, the patient is prone to reoccurring autoimmunity at the node of Ranvier, as she suffers from further autoimmune conditions. AN with anti-neurofascin antibodies has been associated with certain HLA alleles (24, 25), but genetic associations are unknown for other (para)nodal targets, and HLA has not been tested in our patient. Moreover, the damage caused by anti-contactin-1 in the first place could make the paranode more accessible to immune response and therefore trigger secondary autoimmunity against its interaction partner Caspr-1.

Concerning the IgG subclass, we note differences in the course of the disease. With anti-contactin-1 IgG2, the onset and relapse were acute, mimicking GBS. Electroneurography revealed a conduction block, but electromyography did not show signs of chronic damage and motor symptoms were quickly reversible, with the patient achieving full remission (see **Figure 2**). When switching to Caspr-1 IgG4, on the other hand, relapse was slower, but distal tetraparesis was more severe and persisting. Application of rituximab was necessary for 12 months before achieving serological remission and clinical stabilization, with residual axonal damage. Thus, contactin-1 IgG2 probably caused a reversible conduction block, whereas Caspr-1 IgG4 induced secondary axonal damage. Most recently, subclass switch in contactin-1-related AN has also been proposed to play a role in MGN and was related to the acute phase, with histopathological evidence both in nerve and renal biopsies (22). Thus, our case is in line with previous reports of IgG 1-3 being associated with acute and reversible disease and IgG4 with chronic forms of AN and renal pathology (2, 7, 14, 22, 23, 26).

For this patient, the contactin-1 titer was used for follow-up and initially corresponded to the severity of the symptoms as typical for AN (7, 11). Relying on anti-contactin-1 titer only was still misleading upon target antigen switch. Assessing binding to teased fibers with persisting paranodal reactivity made us screen for further target antigens and identify anti-Caspr-1 as a second culprit. Furthermore, we demonstrate the necessity for a long-term follow-up of AN patients, as they might relapse during the course. Thus, we propose to conduct clinical and serological follow-up including testing for the known antibody via ELISA or cell-based assay including titer assessment in a 6-month interval after remission, with a possible extent to 1-year intervals after long-term stabilization. Upon early signs of relapse, testing should be repeated immediately. In AN patients who show clinical deterioration despite serological remission, we strongly recommend screening for further paranodal reactivity using non-target-specific, tissue-based assays like teased fibers binding assay and excluding further known anti-(para)nodal antibodies by CBA and ELISA.

Neurofilament light chain is a ubiquitous marker for axonal damage that has been described as a marker for acuteness, severity, and outcome for inflammatory neuropathies such as GBS and CIDP and recently also for AN (7, 11, 27, 28). Here we report highly elevated sNFL levels during the acute phase of IgG2-associated AN that normalize upon remission. Interestingly, the sNFL levels also rise during relapse with antigen and subclass switch to IgG4, but with a delay of several months, indicating that Caspr-1 IgG4-related axonal damage might be secondary. This case and previous reports (7, 29) strengthen the hypothesis that, in immune-mediated neuropathies and especially AN, the sNFL levels reflect disease activity rather than outcome and are reversible, in contrast to neurodegenerative disease where high levels are linked to a poor outcome (30). They might still not be as immediate as the titer, possibly depending on the underlying pathomechanism.

In conclusion, this case report highlights the importance of assessing the serostatus for the diagnosis, therapy decisions, and follow-up of patients with AN, including the re-evaluation of switching target antigens and subclasses in the course.

## Data Availability

The raw data supporting the conclusions of this article will be made available by the authors, without undue reservation.
